# Freestanding bacterial cellulose-graphene oxide composite membranes with high mechanical strength for selective ion permeation

**DOI:** 10.1038/srep33185

**Published:** 2016-09-12

**Authors:** Qile Fang, Xufeng Zhou, Wei Deng, Zhi Zheng, Zhaoping Liu

**Affiliations:** 1 Ningbo Institute of Materials Technology and Engineering, Chinese Academy of Sciences, Ningbo 315201, P.R. China

## Abstract

Graphene oxide (GO) based membranes have been widely applied in molecular separation based on the size exclusion effect of the nanochannels formed by stacked GO sheets. However, it’s still a challenge to prepare a freestanding GO-based membrane with high mechanical strength and structural stability which is prerequisite for separation application in aqueous solution. Here, a freestanding composite membrane based on bacterial cellulose (BC) and GO is designed and prepared. BC network provides a porous skeleton to spread GO sheets and uniformly incorporates into the GO layers, which endows the BC + GO composite membrane with well water-stability, excellent tensile strength, as well as improved toughness, guaranteeing its separation applicability in water environment. The resulting BC + GO membrane exhibits obviously discrepant permeation properties for different inorganic/organic ions with different size, and in particular, it can quickly separate ions in nano-scale from angstrom-scale. Therefore, this novel composite membrane is considered to be a promising candidate in the applications of water purification, food industry, biomedicine, and pharmaceutical and fuel separation.

Extraordinary molecular separation properties of graphene-based membranes have recently triggered a huge surge of interest due to their good flexibility and precise sieving^1^,^2^,^3^,^4^. Two strategies have been explored to use graphene nanomaterials in membrane processes: nanoporous graphene membrane and stacked graphene oxide (GO) membrane^5^,^6^. Compared with nanoporous graphene membranes^7^,^8^, separation membranes produced from non-porous GO sheets are much cheaper and more easily achievable on a large scale, which realize ion/molecule rejection mainly through the size exclusion of the nanochannels formed by stacked GO sheets^9^,^10^,^11^. These intrinsic and unique channels can be artificially regulated or designed and offer a wide variety of applications for small molecule separation of gas mixtures or aqueous solution^12^,^13^,^14^. Vacuum filtration is the commonly used method to fabricate stacked GO membranes, due to its simplicity and low-cost, which realizes oriented stacking of GO sheets on the surface of a membrane substrate^15^,^16^.

For the sake of fast permeation of the transferable ions/molecules, thin membranes with precise sieving are desirable, generally the thinner the better^17^. However, an ultrathin GO membrane is difficult to be prepared at free-standing state, and it’s a big challenge to peel off such ultrathin membrane from the substrate of filtration equipment^18^,^19^. Usually it is left and covered on the porous substrate which might weaken the performance of the membrane in some cases. Besides, pure free-standing GO membranes usually suffer from low structural stability and are difficult to use in practical applications, especially in aqueous environment for ion/molecule separation^14^,^20^. The GO membranes prepared by vacuum filtration lack sufficient bonding between GO nanosheets, which are likely to dissolve in water again once they contact with aqueous solution. Recently, Huang and his co-works have synthesized a stable GO membrane using anodized aluminium oxide as filter discs, where the multivalent metal cations coming from the filter discs could be introduced unintentionally and crosslink partially negatively charged GO sheets during the synthesis of GO membrane and significantly increased its stability in aqueous solution^21^. Such crosslink effect is thought to be an effective way to enhance the structural stability of GO membranes and render them applicable in aqueous environment.

Apart from the property of structural stability, a separation membrane is required, in some circumstances, to possess a certain degree of mechanical strength to withstand external pressure, such as in the case of backwashing of reverse osmosis membrane. Researches showed that modification of graphene-based membranes via a molecular glue (polymer) could dramatically improve their mechanical strength or toughness^22^,^23^,^24^. However, besides their mechanical properties or electrical conductivity, the sieving efficiency of such nano-composite membranes was seldom concerned. Partial of the nanochannels between stacked GO sheets may be blocked by the intercalated nano-components. Hence, how to prepare a free-standing GO-based membrane with good structural stability in aqueous environment, good mechanical strength and excellent separation performance for different ions/molecules simultaneously is still a driven goal in this novel separation membrane field.

Herein, inspired by the synergistic effect of the composite GO membrane with another component, we attempted to search for a strong two dimensional (2D) network intercalated into the layered GO membrane, in order to improve its mechanical strength while still maintaining good separation performance of the resulted GO-based membrane. Bacterial cellulose (BC), a typical biomass material and readily available at low cost, is consisted of interconnected networks of cellulose nanofibers that aggregate by supra-molecular organization of poly-glucan (C_6_H_10_O_5_)_*n*_ chains^25^,^26^. Compared with plant cellulose, BC possesses higher purity, crystallinity, water-holding capacity and mechanical strength^27^,^28^,^29^. In this study, the mixture of commercial BC and GO nanosheets were vacuum filtrated to form a free-standing composite BC + GO membrane (as shown in Fig. 1) in which GO nanosheets spread on porous BC skeleton. This novel composite membrane presents well water-stability, excellent tensile strength, as well as improved toughness. Of great importance is that it exhibits obviously discrepant permeation properties for different inorganic/organic ions with different size. These properties make the composite membrane a promising candidate in the applications such as water purification (e. g., decolorization of dye wastewater, bacterial filtration), food industry (e. g., decolorization or concentration of beverage/wine), pharmaceutical and fuel separation (e. g., reagent or protein purification, paraffin separation in crude oil treatment), and biomedicine (e.g., artificial kidneys and dialysis) that require precise separation of large and small molecules/ions^14^.

## Results and Discussion

### Preparation of BC + GO membranes

BC produced from bacteria is in form of macroscopic hydrogel pellicle with high structural strength, which is hard for GO nanosheets to incorporate into its network. Actually, BC is known as an insoluble compound to all common solvents, including water, because of the strong hydrogen bond between their abundant –OH groups on the BC chains^30^. In order to obtain homogenous mixture of BC and GO, we smashed the BC hydrogel into micro-hydrogel with a mincer and freeze-dried it as shown in Supplementary Fig. S1a. It can be seen from the scanning electron microscopy (SEM) image in Supplementary Fig. S1b that the treated BC displays a well network structure constituted of numerous fibers entangling together. In order to obtain a stable BC micro-network dispersion, we chose a highly polar solvent of formamide which was thought to have stronger hydrogen bonding with –OH on BC chains than the interaction between –OH themselves^31^. What’s more, formamide is also miscible with water. The comparison of solubility of BC in formamide and water clearly indicates the advantage of formamide in forming homogeneous dispersion of BC over water (Supplementary Fig. S1c). It’s obvious that BC presents stable colloid property in formamide with homogeneous Tyndall phenomenon, while the water solution of BC shows remarkable inhomogeneity.

The preparation of BC + GO membranes is simply realized by the filtration of the mixed solution of BC (formamide solution) and GO (aqueous solution). As shown in Fig. 2a, BC has a strong tendency of combination with GO nanosheets that the BC + GO mixed solution presents stratification after standing. BC extracts GO from the water phase ( mL) to formamide phase (5 mL) although water and formamide are intermiscible. It further implies that the solvent of formamide chosen in this study is an ideal solvent for BC, which even has higher intermiscibility with BC than water. In the BC + GO mixure before filtration, a well dispersive BC micro-network can be clearly observed in the TEM image in Fig. 2b where entangled BC nano-fibers spread evenly on GO sheets, fully guaranteeing the microscopic incorporation of BC and GO. A typical photograph of the as-prepared BC + GO membrane is shown in Fig. 2c, which reveals excellent flexibility. The graph also shows that it is easy to detach the BC + GO membrane from the underlying substrate membrane using a tweezers to get a free-standing membrane, which is more operable than that of pure GO membrane which firmly adheres to the substrate. This simple formation process makes it easy to prepare an integrated membrane in large scale (Fig. 2d). Besides, the resulting BC + GO membrane can be arbitrarily folded without any structure damage (Fig. 2e), preliminarily exhibiting its well tenacity. Generally, the preparation of pure GO membrane through filtration is time-consuming. For instance, the duration of the whole filtration process in Lee *et al*.’s research to fabricate GO papers with different thickness took 6 hour to 1 week^32^. In this study, the strong interaction between BC and GO and obstruction of dense restacking of GO nanosheets by porous BC network largely reduce the filtration time, but the duration time still increased exponentially with the increasing GO content, as displayed in Fig. 2f. BC + GO membranes with low GO ratio are semitransparent, yet the light transmittance of that with high GO ratio is very low, which means the transparency degree of the BC + GO membranes decreases when GO content increases (Fig. 2g).

### Structural and mechanical properties of BC + GO membranes

**Figure 1:**
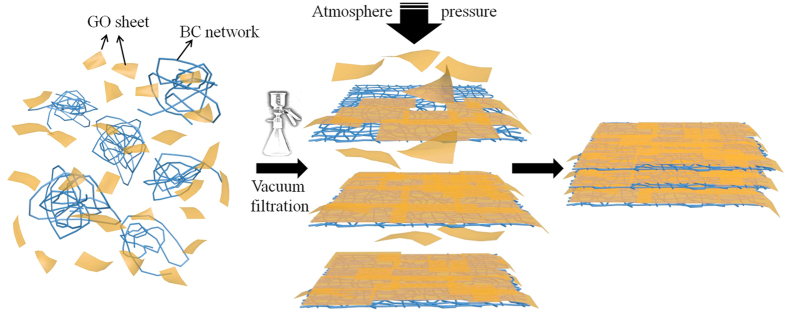
Schematic illustration of the assembly procedure of free-standing BC + GO composite membrane.

**Figure 2:**
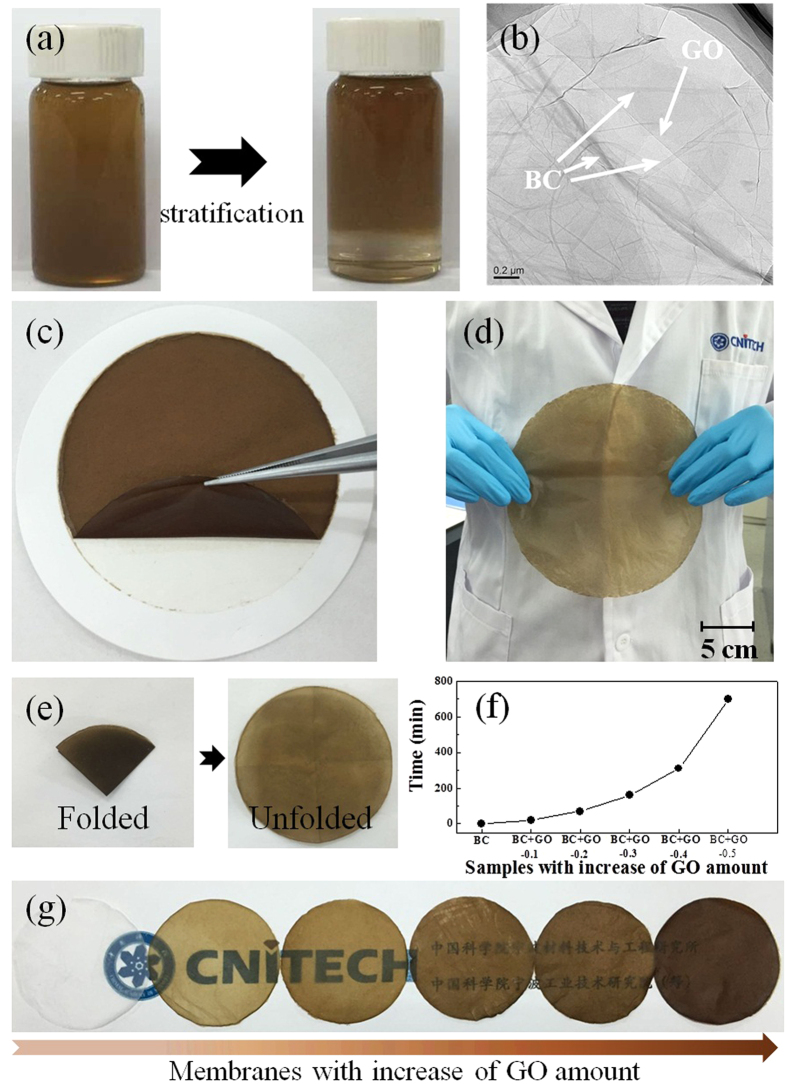
(**a**) Photographs of the mixed solution of BC (formamide, 15 mL) and GO (DI water, 5 mL) before and after standing; (**b**) TEM image of BC and GO mixture from (**a**); (**c**) photograph showing peeling off the BC + GO membrane from the substrate; (**d**) photograph of a large BC + GO membrane having a diameter of 20 cm ; (**e**) photographs of a BC + GO membrane in folded and unfolded state; (**f**) time needed for the filtration process to prepare BC + GO membranes with different GO amounts; (**g**) photograph showing the change of the transparency degree of BC + GO membranes with increase of the GO component.

**Figure 3:**
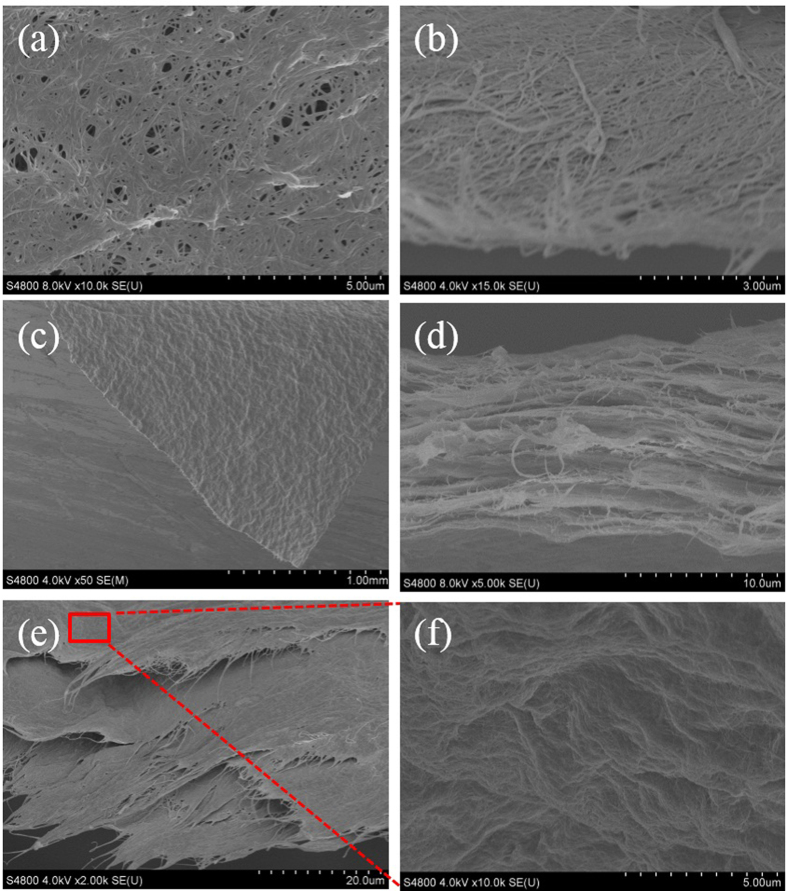
SEM images of (**a,b**) pure BC membrane with low BC loading; (**c**) surface of BC + GO membrane; (**d**) cross-section of BC + GO membrane; (**e**) oblique cross-section of BC + GO membrane; (**f**) the designated area in (**e**).

Figure. 3 provides the surface and cross-section SEM images of pure BC and BC + GO membranes. BC micro-network would be compressed into a plane porous network under vacuum filtration as shown in SEM images in Fig. 3a,b. Pure BC membrane with network structure has randomly distributed pores in the size range of tens to hundreds of nanometers, which will not limit the transfer of ions/molecules and can be served as skeleton for GO deposition as expected. After combining with GO nanosheets, an uneven surface is observed from the top-view SEM image of BC + GO membrane (Fig. 3c), indicating that GO nanosheets have been tightly covered on the BC skeleton. Except for the physical contact between BC and GO caused by the filtration process, hydrogen bonds between the –OH from BC and oxygen-containing groups on GO surface can be a main force to drive the close adhesion of GO nanosheets and BC network support. The cross-sectional image of BC + GO membrane as shown in Fig. 3d presents loosely stacked layered structure, which is different from the compact layers in pure GO membrane (Supplementary Fig. S2a). Besides, the pulling-out of some BC fibers is clearly shown between the stacked GO sheets in BC + GO membrane. These observations imply that BC network has been successfully and uniformly intercalated into the GO stacking and served as skeleton for GO sheets deposition, strongly agreeing with the schematic illustration in Fig. 1. Giving an insight into the oblique cross-section of BC + GO membrane in Fig. 3e, more remarkable lamination of BC skeleton + GO stacking layers can be observed. With a magnified observation of the staggered layers in Fig. 3e, every sublayer exhibits an out-of-flatness surface as shown in Fig. 3f where numerous GO nanosheets deposit and stack on the BC network skeleton (more SEM images is shown in Supplementary Fig. S3). The morphology of the sublayers of BC + GO membrane is in significant contrast to that of the pure BC membrane in Fig. 3a, and there are no visible holes on the sublayers which might be due to the tight coverage of BC network by GO nanosheets (whose size is about 1~2 μm, Supplementary Fig. S4).

**Figure 4:**
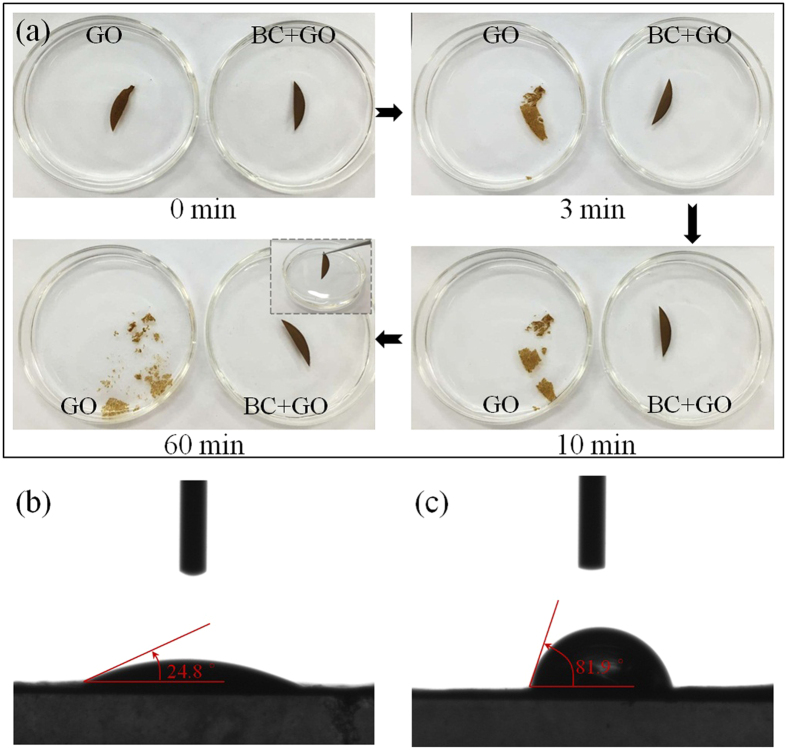
(**a**) Photographs of the pure GO and BC + GO membranes soaked in water for different time, and the inset picture displays BC + GO membrane held with a tweezers; (**b**,**c**) the static contact angles of pure GO membrane (**b**) and BC + GO membrane (**c**).

The structural stability of the BC + GO membrane was examined by soaking it in water, and pure GO membrane was employed as a comparison (Fig. 4a). GO membrane begins to break up in water within 3 min and completely disintegrates after 60 min without any mechanical agitation. In sharp contrast, the BC + GO membrane maintains an intact membrane structure during the whole process, which can even be handled with tweezers without structural damage (as shown in the inset photo in Fig. 4a). Apparently, because of the strong interaction between GO sheets and BC skeleton, it protects BC + GO membrane to suffer from hydration, swelling and subsequent disintegration which occurred on the pure GO membrane^21^. Therefore, the BC + GO membrane here can be used in water environment and withstand certain external forces during permeation operation. The hydrophilic-hydrophobic property of the pure GO and BC + GO membranes was evaluated by measuring their water contact angles. As shown in Fig. 4b,c, the contact angle increases from 24.8° for the pure GO membrane to 81.9° for the BC + GO membrane, indicating that the composite membrane surface displays a higher hydrophobic nature. This can be understood in light of the apparent roughness of the BC + GO membrane compared to pure GO membrane, which can be obviously distinguished from the surface SEM images of BC + GO (Fig. 3c) and pure GO (Supplementary Fig. S2b) membranes. The increased contact angle nicely accounts for the restraint of hydration and swelling of the composite membrane to prevent its disintegration in aqueous solution, however it may increase the risk of membrane fouling in application due to the increased surface roughness, which needs to be further improved^33^.

**Figure 5:**
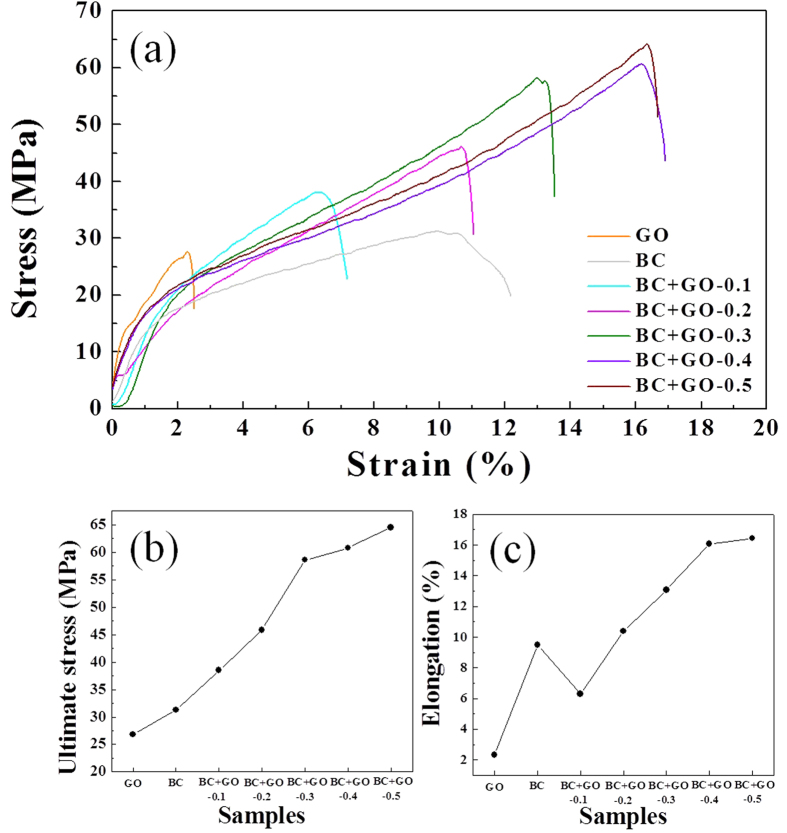
Mechanical properties of the pure GO membrane, pure BC membrane, and BC + GO membranes with different GO amounts. (**a**) The stress-strain curves, (**b**) the change of ultimate stress, and (**c**) the change of elongation.

Typical stress-strain curves were measured to evaluate the mechanical properties of the membranes as shown in Fig. 5a. Pure GO membrane has the lowest tensile strength with the ultimate stress of 26.8 MPa, while that of pure BC membrane slightly increases to 31.3 MPa. The BC + GO membranes have remarkable enhancement of the tensile strength comparing with either GO or BC membranes, and their tensile strength increases continuously with the increase of GO amounts (Fig. 5b). In particular, the sample of BC + GO-0.5 reaches an ultimate stress of 64.5 MPa, more than two times of that of pure GO or BC membranes. The combination of GO and BC can also enhance the elongation at break of the composite membranes as shown in Fig. 5c. It can be observed that the membrane prepared from pure BC possesses a good ductility (elongation of 9.5%), which is due to the tight intertexture of numerous cellulose nanofibers. The elongation increases gradually with the addition of GO, but ruling out an exception of the sample of BC + GO − 0.1. Such enhancement can be attributed to the effective load transfer between the components of BC and GO through their strong interfacial interactions^34^. BC is in rich of hydroxyls which can form strong hydrogen bonds with the oxygen-containing groups on GO surface, similar to another type of graphene-based artificial nacre where the synergistic effect played a key role for the mechanical properties’ improvement of the composite membranes^35^,^36^. However, with a low addition amount of GO (BC + GO − 0.1), the interfacial interaction between BC and GO is not strong enough to offset the weakened interaction between the cellulose nanofibers themselves, subsequently exhibiting a decreasing elongation compared with pure BC membrane.

### Ion permeation performance of BC + GO membranes

**Figure 6:**
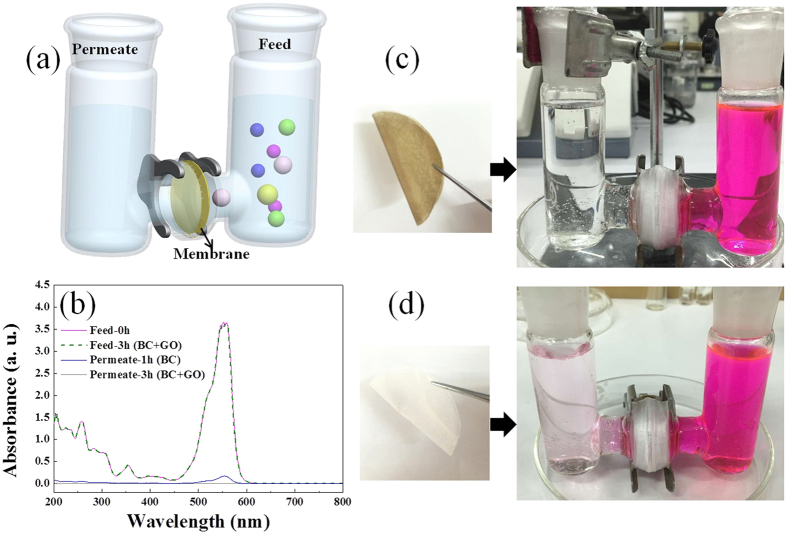
(**a**) Schematic of the experimental apparatus used in permeation test; (**b**) UV-vis absorption spectra of RhB in both of the feed and permeate solutions before and after permeation through BC + GO-0.3 and pure BC membranes at the initial feed solution concentration of 50 mg L^−1^; (**c**,**d**) photographs of the used membranes and apparatus during the permeation process, (**c**) BC + GO membrane after 3 h of permeation and (**d**) pure BC membrane after 1 h of permeation.

The BC + GO membranes were tested for their ion permeation performance using the experimental apparatus as shown in Fig. 6a. In such apparatus which is divided by the test membrane into two independent chambers, the hydrostatic pressure resulted from slight liquid level change during the experiment process can be neglected due to the relatively short permeation time (the longest of 3 h)^1^. Organic cation of rhodamin B (RhB, with size of 1.8 × 1.4 nm) was firstly selected as the test ion for its permeation property through BC + GO membrane. At the initial concentration of 50 mg mL^−1^ for the feed solution, the UV-vis absorption spectra of both the feed and permeate solutions were measured before and after 3 h of permeation (Fig. 6b). Notably, there is no color change in the feed chamber, and the UV-vis spectra of RhB solution at 0 h and 3 h overlap well. Simultaneously, there are no organic ions detected in the permeate solution after 3 h, which can be visually observed (Fig. 6c) and further verified by the UV-vis absorption spectrum. In contrast, RhB can quickly pass through the membrane prepared from pure BC only after 1 h of permeation. The initially colorless solution in the permeate chamber turned rose red within a short time of permeation (Fig. 6d), and characteristic absorption peak of RhB at 554 nm was detected in its UV-vis absorption spectrum (Fig. 6b). Organic anion of methyl orange (MO, with size of 1.54 × 0.48 nm) behaved similar permeation property on BC + GO membrane as that of RhB, which also can’t diffuse through it (Supplementary Fig. S5). These results indicate that the BC + GO membrane can effectively reject the diffusion of organic ions, and GO component makes the main contribution for ions interception. Although GO surface presents electronegativity, the electrostatic attraction between GO and the diffusing ions can be excluded for their effect on the ion interception by BC + GO membrane, on account of that both of cationic RhB and anionic MO have been rejected to permeate and the concentration of feed solution remain unchanged, which further suggests that the size exclusion comes from the nanochannels of stacked GO sheets should be the main interception mechanism. The average size of the nanocapillaries of BC + GO membrane is smaller than that of organic dye molecules, which leads to that RhB and MO can’t transfer through the nanochannels formed by stacked GO sheets and diffuse to the other side of the membrane. Therefore, physical sieving by nanochannels is believed as the dominant factor in retention of large organic molecules^9^,^18^,^19^. But it’s undeniable that electrostatic repulsion might play another role in rejecting negatively charged molecule of MO^18^.

**Figure 7:**
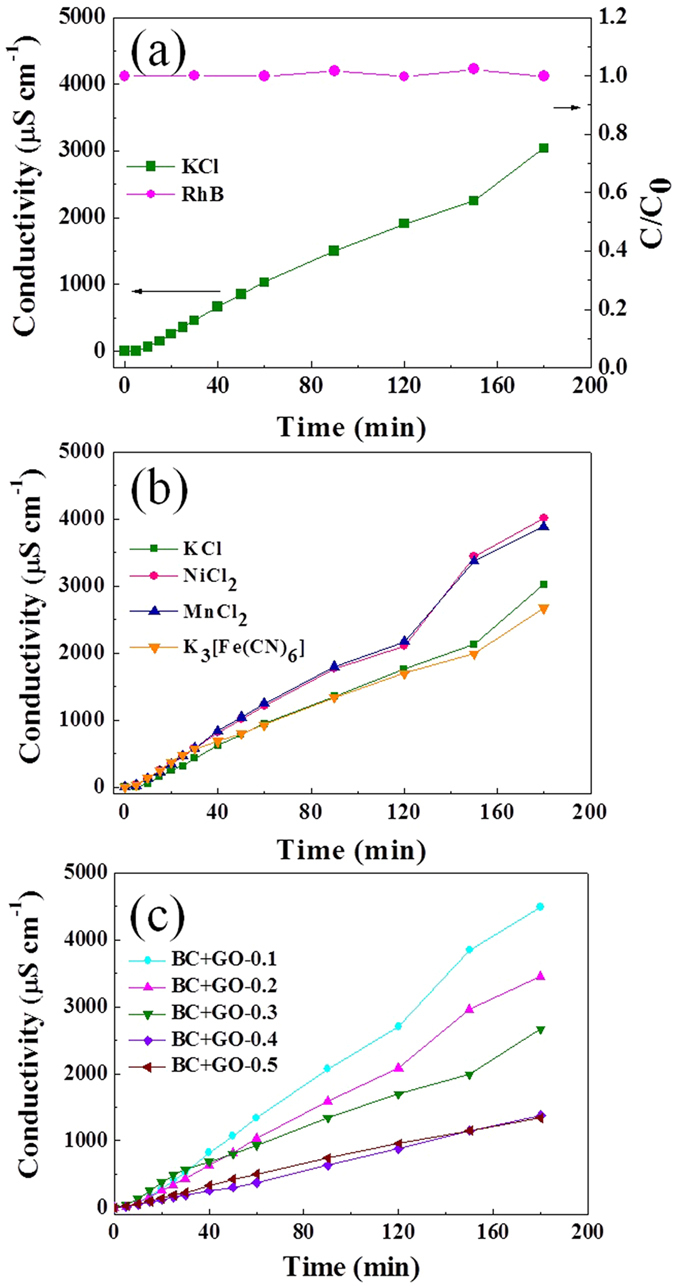
(**a**) The permeation process of a mixture of KCl (0.2 M) and RhB (50 mg L^−1^) based on BC + GO-0.3 membrane. The left ordinate represents the changes of permeate solution conductivity and the right ordinate represents the concentration (*C/C*_*o*_) changes of RhB in feed solution; (**b**) The permeation process of different inorganic ions (0.2 M) through BC + GO-0.3 membrane; (**c**) The permeation process of K_3_[Fe(CN)_6_] (0.2 M) based on BC + GO membranes with different GO contents.

Then, a permeation process of mixed KCl (0.2 M) and RhB (50 mg mL^−1^) solution was carried out on BC + GO membrane, and KCl transport was probed by monitoring the conductivity of the solution in the permeate chamber. As presented in Fig. 7a, the conductivity changes slightly within the initial 15 min, and it increases drastically in linear with time in the following time. Meanwhile, there is no color change in the permeate chamber and the relative concentration (*C/C*_*o*_) of RhB detected in the feed chamber keeps constant at 1. Consequently, it can be concluded that ions of K^+^ and Cl^−^ with small size (hydrated radii <4 Å) can easily and quickly diffuse through the composite membrane while RhB with large ion size (hydrated radius >1 nm) can’t. The sizes of different ions are listed in Supplementary Table S1. This evidently manifests that the BC + GO membrane prepared in this study have selective ion permeation property according to the size of ions, which can be used for small molecule/ion separation or size grading in a certain range. The slow slope segment in the initial stage of KCl permeation in Fig. 7a means that the wetting of BC + GO membrane in solution can be finished in a short time and the nanochannels in the membrane can quickly swell to allow fast migration of small hydrated ions.

A series of similar ion permeation tests were also conducted for several salt solutions (0.2 M) with different ion sizes, and their permeation curves are presented in Fig. 7b. Obviously, all the tested ions have hydrated radii smaller than 1 nm can permeate through the BC + GO membrane, and no rejection of ions with size grading can be observed. Theoretically, the molar conductivity of trivalent [Fe(CN)_6_]^3−^ is much higher than monovalent Cl^−^ at the same temperature, however, conductivities of the solutions in the permeate chambers for both KCl and K_3_[Fe(CN)_6_] at the same feed concentration of 0.2 M are almost the same within the whole permeation time range, and the conductivity of permeate K_3_[Fe(CN)_6_] is even lower than KCl at the later stage of permeation. This result powerfully demonstrates that the permeation rate of [Fe(CN)_6_]^3−^ with larger hydrated ion radius (4.75 Å) through the composite BC + GO membrane is lower compared with smaller ion of Cl^−^ (3.32 Å). It is speculated that the dimension of partial of the nanochannels formed by stacked GO sheets is not large enough to allow penetration of [Fe(CN)_6_]^3−^, while the rest are still dimensionally matching. What’s more, it’s thought that anions permeate through the nanocapillaries more easily than cations do during the process of iontophoretic injection because of the strong repulsive forces between the anions and the functional groups of GO^3^, hence, the higher the valence is, the stronger the repulsion is, and the faster the diffusion is during the membrane. However, in fact it is quite the opposite that the penetration of [Fe(CN)_6_]^3−^ is slower than Cl^−^, which is another proof of size exclusion as mentioned above. Similarly, one Ni^2+^ migrates with two Cl^−^, and the conductivity of the NiCl_2_ permeate chamber will be nearly twice of that of KCl if Ni^2+^ and K^+^ have the same diffusion rate through the membrane. Actually, the measured conductivity of NiCl_2_ is much less than two times higher than that of KCl, which implies that the channels in BC + GO membrane also have some inhibition effect for diffusion of Ni^2+^. Herein, electrostatic attraction between Ni^2+^ and the oxygen-containing groups of GO occurred during the process of iontophoretic injection might be another factor contributing for its lower permeation rate^3^. Heavy-metal salts of NiCl_2_ and MnCl_2_ show identical permeation curves. It’s known that Ni^2+^ (4.04 Å) and Mn^2+^ (4.38 Å) possess similar hydrated radii, hence there is no detectable diffusion difference between the two ions. In brief, although the BC + GO membrane can’t further precisely sieve the ions in angstrom-scale as the GO membrane reported by Joshi, *et al*. that can block all the solutes with hydrated radii larger than 4.5 Å^1^, this composite membrane still exhibit selective permeation for small ions with different hydrated radii. The smaller the size of the ions is, the faster the permeation will be.

It’s worth mentioning that the permeation curves of KCl in Fig. 7a,b are almost the same, suggesting that the presence of RhB in the mixed feed solution (Fig. 6d) didn’t affect the permeation property of KCl, which further rules out the possible adsorption of RhB onto the GO nanosheets in BC + GO membrane. If there is any RhB adsorbed on the membrane surface, it will block the entrance formed by adjacent GO sheets and prevent the access of KCl. π-π interaction has been considered as an interaction force for the adsorption of organic dyes (with benzene ring) onto graphene-based materials^37^, however, in this case, it can be neglected since that there are very small amount of GO covered on the membrane surface and the weak π contribution from GO can also be negligible compared with graphene. This phenomenon further demonstrates that the rejection of organic ions with large size is mainly ascribed to the size exclusion of GO channels rather than adsorption.

As designed in advance, GO is verified to be the critical constituent in BC + GO membrane that determines its separation performance, therefore, we further assessed the permeation properties of BC + GO membranes with different amount of GO using K_3_[Fe(CN)_6_] as the test ions. As shown in Fig. 7c, after 3 h of permeation, significant difference can be observed in the permeation curves of the series of BC + GO membranes, whose permeation rate decreases remarkably with the increase of GO amount. The increasing content of GO will certainly increase the thickness of final BC + GO membrane, which can be seen from the SEM images in Supplementary Fig. S6, and it’s not difficult to understand that the thickening of diffusion path via GO barrier will hamper the small ions in the angstrom-scale to migrate to the other side of the permeation membrane. Whereas, BC + GO − 0.4 and BC + GO − 0.5 have similar permeation curves, implying that the diffusion rate will not be influenced by the membrane thickness any more when the GO component increases to a threshold amount in the composite membrane, but is only determined by the ion concentration in feed solution. From a general view, the permeation rate of small inorganic ions in this study is much faster than that reported on pure GO membrane by an order of magnitude, based on the rough calculation of permeate conductivity^3^. This might be ascribed to that BC network has no barrier effect for ion transfer, and the amount of effective GO nanosheets in the composite membrane is much less compared to a pure GO membrane, thus the shorter diffusion path results in faster permeation. Just as shown in Fig. 4a, the property of easy disintegration of pure GO membrane in aqueous solution makes it hard for us to carry out its permeation test for comparison in this work’s experimental apparatus without any support, which further illustrates that the stability of GO membrane in water is a prerequisite for their application in aqueous solution, as reported by Yeh, *et al*.^21^.

**Figure 8:**
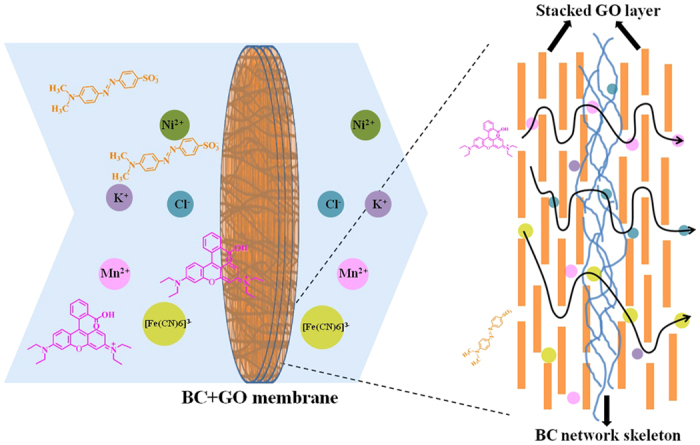
Schematic of the permeation process of different organic or inorganic ions through BC + GO membrane.

The permeation process and mechanism are simply summarized in the schematic diagram of Fig. 8. The component of BC in this composite membrane acts as supporting scaffold for GO deposition to make the composite membrane a free-standing one with high mechanical strength and water stability, guaranteeing its sustained operation in practical permeation process, and its open framework will not impede ion diffusion. It is the GO component that really plays the role of selective ion permeation. The channels formed by stacked GO sheets intrinsically determine if the ions can transfer through the composite membrane^4^,^18^,^38^. As shown in Fig. 8, the organic ions with large size in nanoscale (RhB and MO) will be rejected by the GO channels via size exclusion, while all the tested inorganic ions with smaller size (all less than 1 nm), such as K^+^, Ni^2+^, Mn^2+^, Cl^−^ and [Fe(CN)_6_]^3−^, can permeate through it, but their permeation rates decrease with their increasing hydrated radii, due to that partial of the channels still have size exclusion for these small ions. The difference of permeation rates can be further used for selective permeation via proper control of permeation time and GO content in BC + GO membrane.

## Conclusions

In summary, we have developed a high-performance separation membrane based on bacterial cellulose and graphene oxide. The successful dispersion of BC gel using formamide fully guarantees the microscopic incorporation of BC and GO, where BC network acts as supporting skeleton for the spread of GO sheets within the composite membrane. Such synthesis pathway coupled with synergistic effect of two components largely improves the composite membrane’s water stability and mechanical strength compared with pure GO membrane. Of great importance is that the prepared BC + GO membrane in this study can realize selective ion permeation for different inorganic/organic ions with different size and quickly separate ions in nano-scale from angstrom-scale, which has great application potential in the separation field.

## Materials and Methods

### Preparation of GO and BC dispersion

Graphene oxide (GO) dispersion was synthesized from natural graphite powder using a modified Hummers’ method as we reported previously^39^ and GO dispersion was diluted into different concentration as required. Bacterial celluloses (BC) were provided by TianAn Biologic Materials Co., Ltd., China in form of hydrogel pellicles which were purified in the following process. The hydrogel was washed with water and subsequently treated with 2% (w/v) NaOH solution at 60 °C for 24 h, and finally rinsed again with water^40^. The purified BC was minced into microgel and freeze-dried as shown in Supplementary Fig. S1. Then the dried BC was dispersed in formamide with sonication for 2 h at 400 W to prepare stable BC dispersion with concentration of 1 mg mL^−1^.

### Preparation of GO, BC and BC + GO membranes

15 mL BC dispersion (1 mg mL^−1^) was mixed with 5 mL GO dispersion (0, 0.1, 0.2, 0.3, 0.4, 0.5 mg mL^−1^) and sonicated for 10 min. Then the mixture was vacuum filtrated to prepare BC and BC + GO membranes. After filtration, the membrane was peeled off from the substrate film (hybrid fiber microporous film with 0.22 μm pore size) of the filter and dried at ambient conditions. The obtained BC + GO membranes whose thickness were at the range of 11~17μm were referred to as BC + GO − 0.1, BC + GO − 0.2, BC + GO − 0.3, BC + GO − 0.4 and BC + GO − 0.5 according to the concentration of the added GO dispersion. The pure GO membrane was prepared from 40 mL 1 mg mL^−1^ GO dispersion (a similar thickness range to BC + GO membrane) using the same procedure without the addition of BC.

### Characterization

The microstructures of the membranes and their precursors were characterized by a transmission-electron microscope (TEM, Tecnai F20, FEI) and a field emission scanning electron microscopy (FE-SEM, S4800, Hitachi). Static contact angles (SCAs) were obtained using a contact angle meter (OCA20, Data physics) via static sessile drop method. The membranes were cut into a 5 mm × 25 mm strip using a laser cutter for mechanical property test which was conducted on a universal testing machine (5569A, Instron) at a loading rate of 2 mm min^−1^.

### Ion-permeation test

The ion permeation tests were carried out in a made-to-order apparatus as shown in Fig. 6a, which is consisted of two independent tubular chambers. The freestanding membrane was fixed in the middle of the two chambers and clamped with a screw clamp, subsequently wrapped with Parafilm to prevent leakage. The exposed membrane diameter is 2 cm. In a typical test, 35 mL salt solution was injected into one chamber as feed solution and simultaneously 35 mL deionized water was injected into another chamber. Therefore, there was no hydrostatic pressure contributing for permeation, and concentration gradient is thought to be the main driving force. Both in the feed and permeate solution, magnetic stirring was given to avoid possible concentration polarization effect near the membrane. Ion diffusion was monitored by measuring conductivity and/or UV-vis spectra of the solution in feed and/or permeate chambers. The conductivity was detected by a conductivity meter (DDS-307A, INESA) at 25 °C and UV-vis spectra measurement was performed on a UV/vis/NIR spectrophotometer (Lambda 950, PerkinElmer).

## Additional Information

**How to cite this article**: Fang, Q. *et al*. Freestanding bacterial cellulose-graphene oxide composite membranes with high mechanical strength for selective ion permeation. *Sci. Rep.* **6**, 33185; doi: 10.1038/srep33185 (2016).

## Supplementary Material

Supplementary Information
