# Supplementary material for: Freestanding bacterial cellulose-graphene oxide composite membranes with high mechanical strength for selective ion permeation
Source: Sci Rep. 2016 Sep 12;6:33185. doi: 10.1038/srep33185 (PMC5018816; doi:10.1038/srep33185)
Supplement: Supplementary Information [file srep33185-s1.pdf]

Supplementary Information for

Freestanding bacterial cellulose-graphene oxide composite membranes with high mechanical strength for selective ion permeation

Qile Fang, Xufeng Zhou, Wei Deng, Zhi Zheng, and Zhaoping Liu**

Ningbo Institute of Materials Technology and Engineering, Chinese Academy of Sciences, Ningbo 315201, P. R. China.

*Corresponding author E-mail: liuzp@nimte.ac.cn (Z. Liu); zhouxf@nimte.ac.cn (X. Zhou)

The SI file includes:

Figure S1 (a) Photographs displaying the treatment of BC from hydrogel to aerogel; (b) SEM image of BC micro-network structure; (c) Photographs showing the dispersion of BC aerogel in H_2O and formamide.

Figure S2 SEM images of the (a) cross-section and (b) surface of pure GO membrane.

Figure S3 SEM images of the BC+GO membrane, and (b, c) are the designated areas in (a) with higher magnification.

Figure S4 AFM image and corresponding height profile of the prepared GO nanosheets.

Figure S5 (a) Photographs of the apparatus based on BC+GO-0.3 membrane for MO permeation after 3 h; (b) the corresponding UV-vis absorption spectra of MO in the feed and permeate solution.

Figure S6 SEM images of the cross-section of (a) BC+GO-0.1, (b) BC+GO-0.2 and (c) BC+GO-0.3 membranes with their thickness marked.

Table S1 Hydrated radii of inorganic ions and size of organic ions

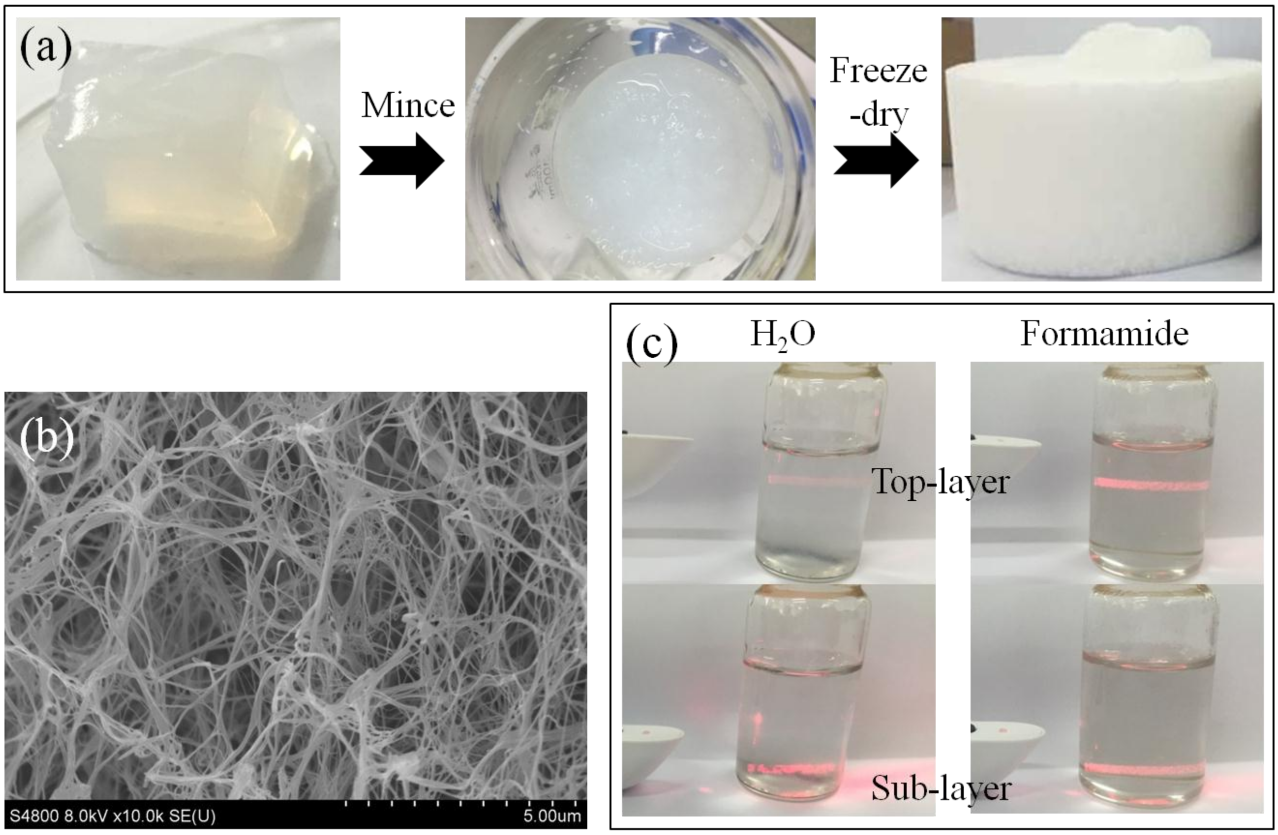

Figure S1. (a) Photographs displaying the treatment of BC from hydrogel to aerogel; (b) SEM image of BC micro-network structure; (c) Photographs showing the dispersion of BC aerogel in H_2O and formamide.

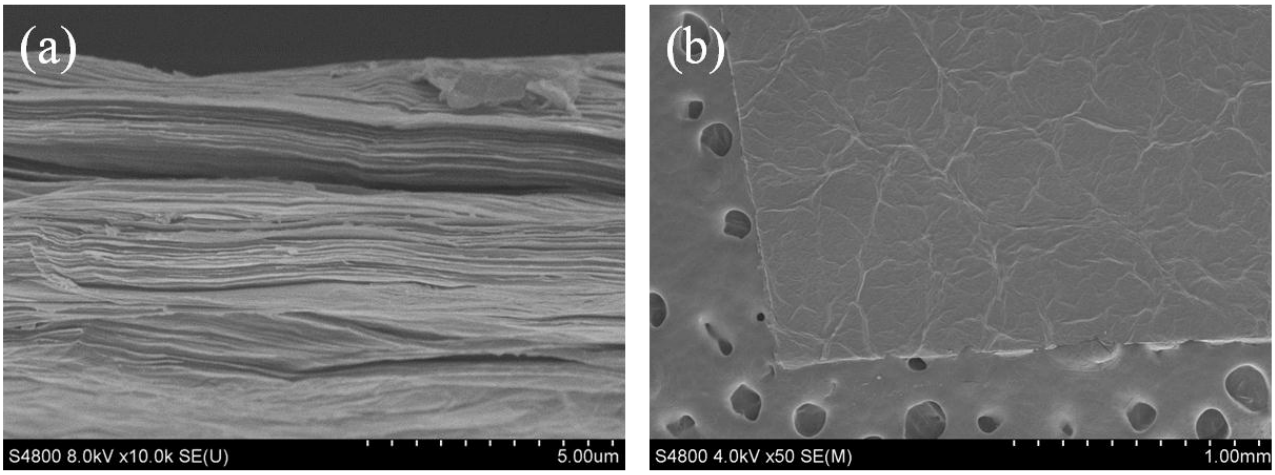

Figure S2. SEM images of the (a) cross-section and (b) surface of pure GO membrane.

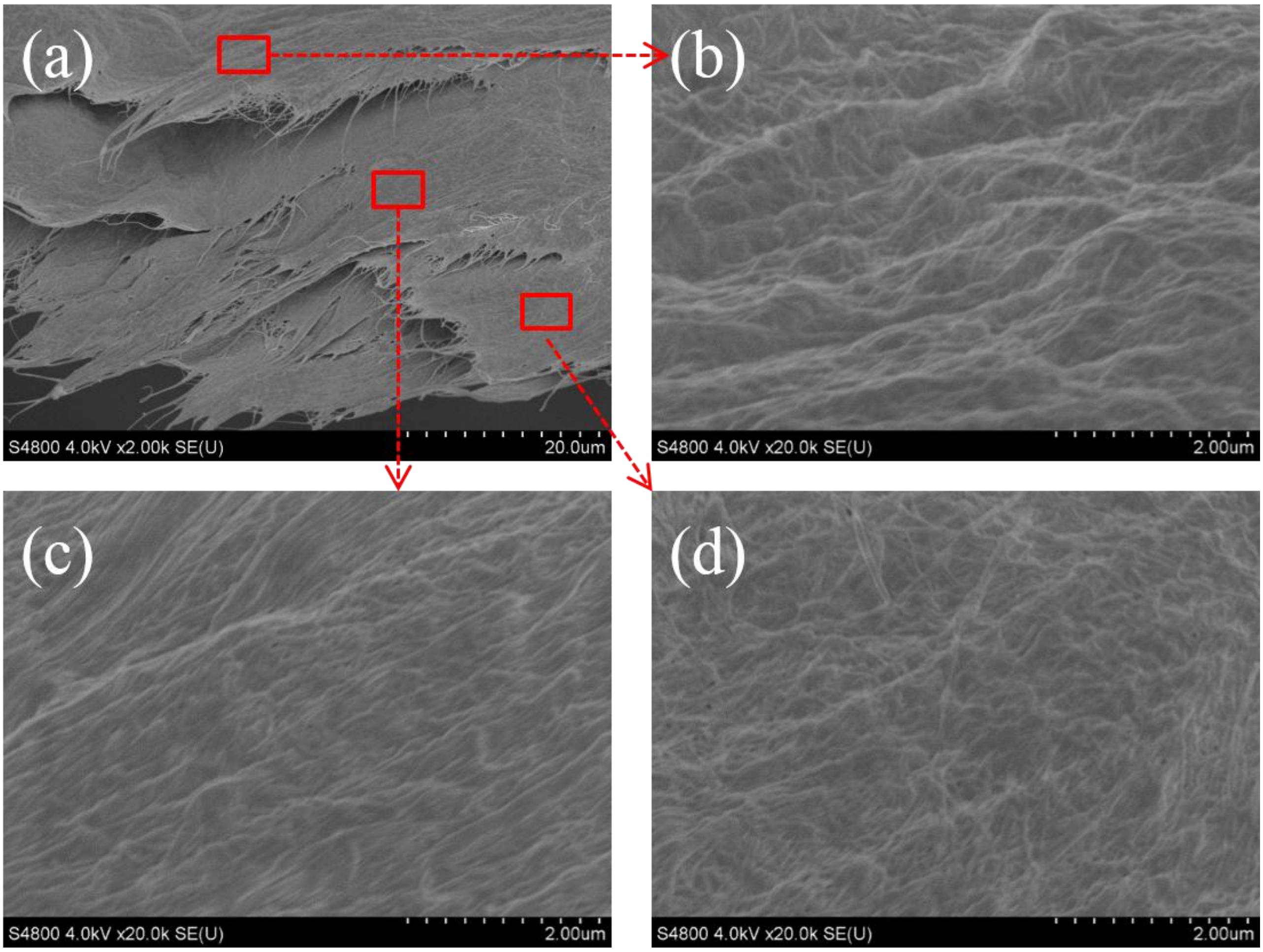

Figure S3. SEM images of the BC+GO membrane, and (b, c, d) are the designated areas in (a) with higher magnification.

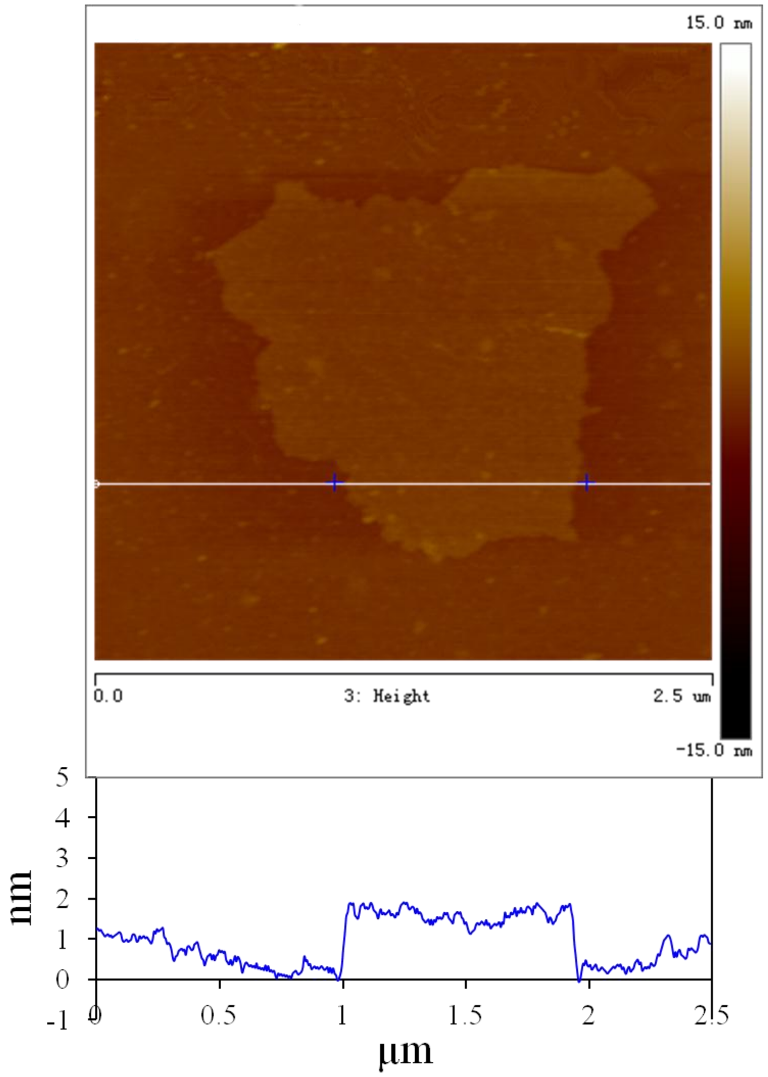

Figure S4. AFM image and corresponding height profile of the prepared GO nanosheets.

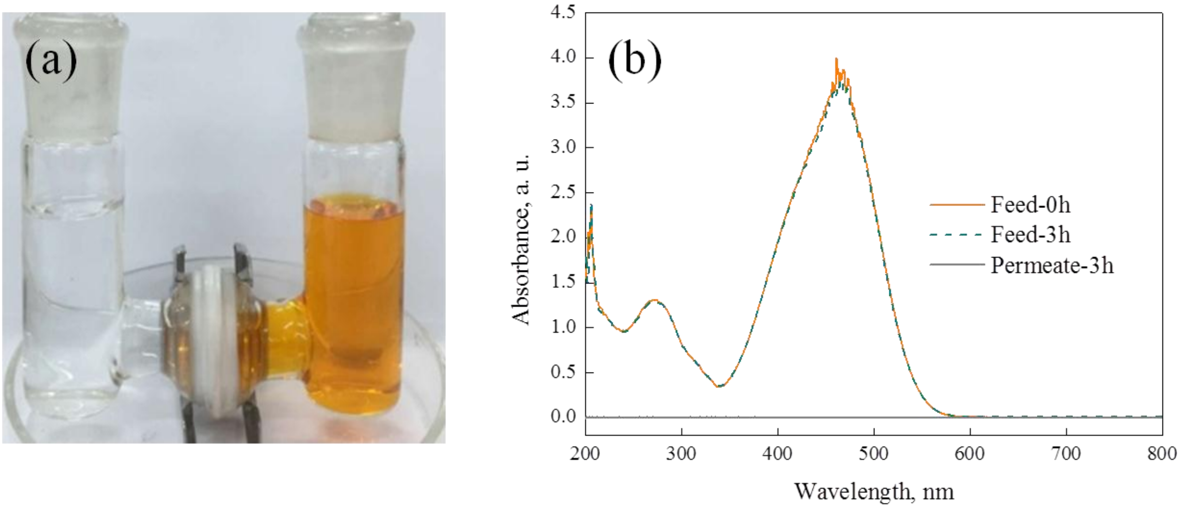

Figure S5. (a) Photographs of the apparatus based on BC+GO-0.3 membrane for MO permeation after 3 h; (b) the corresponding UV-vis absorption spectra of MO in the feed and permeate solution.

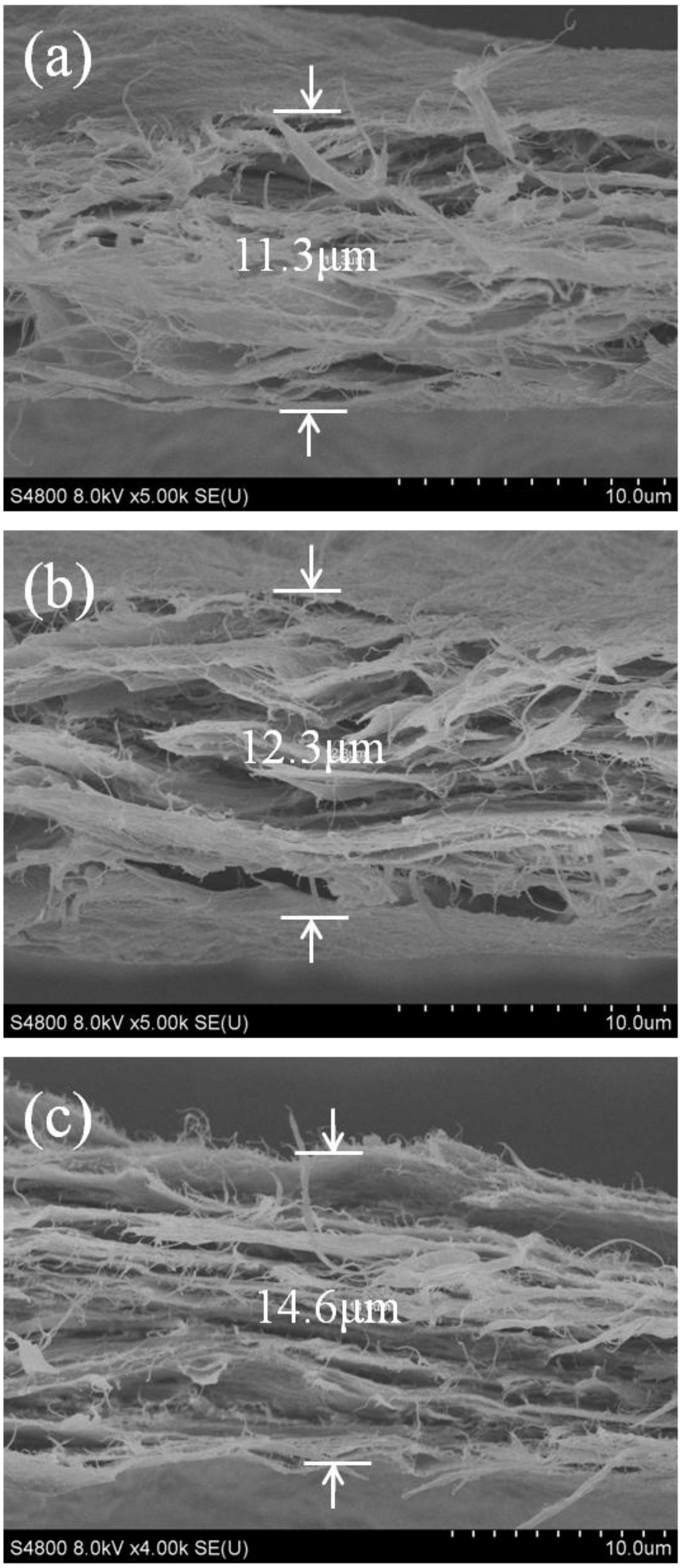

Figure S6. SEM images of the cross-section of (a) BC+GO-0.1, (b) BC+GO-0.2 and (c) BC+GO-0.3 membranes with their thickness marked.

Table S1. Hydrated radii of inorganic ions and size of organic ions

Ion	Hydrated radius, Å
K^+	3.31
Cl^-	3.32
Ni^{2+}	4.04
Mn^{2+}	4.38
$[Fe(CN)_6]^{3-}$	4.75
RhB 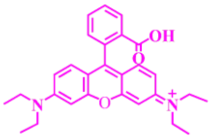	1.8 ×1.4 nm
MO 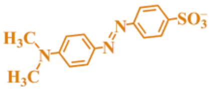	1.54 ×0.48 nm
